# 5-HT_2A_ Receptor Knockout Mice Show Sex-Dependent Differences following Acute Noribogaine Administration

**DOI:** 10.3390/ijms25020687

**Published:** 2024-01-05

**Authors:** Sofía Villalba, Bruno González, Stephanie Junge, Alejandra Bernardi, Joaquín González, Catherine Fagúndez, Pablo Torterolo, Ignacio Carrera, Francisco J. Urbano, Verónica Bisagno

**Affiliations:** 1 Instituto de Investigaciones en Medicina Traslacional, Facultad de Ciencias Biomédicas, CONICET-Universidad Austral, Mariano Acosta 1611, Buenos Aires B1629WWA, Argentina; mvillalba-iimt@austral.edu.ar (S.V.); stephijunge@gmail.com (S.J.);; 2 Departamento de Fisiología, Biología Molecular y Celular Prof. Héctor Maldonado, Instituto de Fisiología, Biología Molecular y Neurociencias (IFIBYNE-CONICET), Facultad de Ciencias Exactas, Universidad de Buenos Aires, Buenos Aires C1428EGA, Argentina; fjurbano@fbmc.fcen.uba.ar; 3 Departamento de Química Orgánica, Facultad de Química, Universidad de la República, Avenida General Flores 2124, Montevideo 11800, Uruguay; brunogonzalez@fq.edu.uy (B.G.); cfagundez19@gmail.com (C.F.); icarrera@fq.edu.uy (I.C.); 4 Departamento de Fisiología, Facultad de Medicina, Universidad de la República, Avenida General Flores 2125, Montevideo 11800, Uruguay; joaqgonzar@gmail.com (J.G.); ptortero@fmed.edu.uy (P.T.)

**Keywords:** psychedelics, noribogaine, serotonin, NMDA, glutamate receptors, immediate early genes (IEG)

## Abstract

Noribogaine (noribo) is the primary metabolite from ibogaine, an atypical psychedelic alkaloid isolated from the root bark of the African shrub *Tabernanthe iboga*. The main objective of this study was to test the hypothesis that molecular, electrophysiological, and behavioral responses of noribo are mediated by the 5-HT_2A_ receptor (5-HT_2A_R) in mice. In that regard, we used male and female, 5-HT_2A_R knockout (KO) and wild type (WT) mice injected with a single noribo dose (10 or 40 mg/kg; i.p.). After 30 min., locomotor activity was recorded followed by mRNA measurements by qPCR (immediate early genes; IEG, glutamate receptors, and 5-HT_2A_R levels) and electrophysiology recordings of layer V pyramidal neurons from the medial prefrontal cortex. Noribo 40 decreased locomotion in male, but not female WT. Sex and genotype differences were observed for IEG and glutamate receptor expression. Expression of 5-HT_2A_R mRNA increased in the mPFC of WT mice following Noribo 10 (males) or Noribo 40 (females). Patch-clamp recordings showed that Noribo 40 reduced the NMDA-mediated postsynaptic current density in mPFC pyramidal neurons only in male WT mice, but no effects were found for either KO males or females. Our results highlight that noribo produces sexually dimorphic effects while the genetic removal of 5HT_2A_R blunted noribo-mediated responses to NMDA synaptic transmission.

## 1. Introduction

The potential use of psychedelic compounds in psychiatry has recently generated an extensive body of work regarding the therapeutic actions of these molecules [1]. Several studies suggest that psychedelic compounds such as psylocibin and MDMA might have a place in the treatment of several psychiatric disorders that represent a huge suffering and economic burden given their difficulty of treatment and high relapse rate. Safety, efficacy, and tolerability were found by clinical trials involving psychedelic-assisted psychotherapy for anxiety and depression [2], PTSD [3], alcohol use [4], and depressive disorders in cancer patients [5]. 

It needs to be noted that unlike many psychiatric medications, this type of treatment requires administration of the compound only once or twice over a few weeks, which are preceded and followed by preparation and integration sessions with a trained therapist. Serotonergic neurotransmission, and particularly the one mediated by 5-HT_2A_ receptors (5-HT_2A_R), has been implicated not only for the sensory and cognitive alterations induced by the acute administration of classical psychedelics but also as a key target for their therapeutic properties [6]. 5-HT_2A_R are densely expressed in thalamocortical presynaptic and postsynaptic terminals in the V layer of the medial prefrontal cortex [7,8,9,10].

Ibogaine is a naturally occurring alkaloid derived from the *Tabernanthe iboga* shrub, which is native to West Africa [11,12,13]. It has been classified as a potent atypical psychedelic drug capable of inducing oneirogenic effects (waking dream-like states) and vivid memory recall. Ibogaine and noribogaine (10-hydroxyibogamine; noribo, the primary active metabolite of ibogaine produced by a CYP2D6-dependent pathway; [14,15]) have gained increasing attention due to their promising “anti-addictive” properties documented in observational and open-label studies in humans and in pre-clinical models [16,17,18,19,20]. In addition, a rapid antidepressant effect in humans after ibogaine administration was described in previous clinical trials, and both ibogaine and noribogaine have shown antidepressant-like effects in rodents [15]. 

Nevertheless, the neurobiological mechanism underlying the antiaddictive and antidepressant effects displayed by ibogaine and noribogaine remain unsolved [13]. Initial receptor-binding studies for ibogaine indicated a polypharmacological profile, with binding to numerous targets at the micromolar range, such as nicotinic acetylcholine receptors (nAChR α3β4), the N-methyl-D-aspartate receptor (NMDAR), kappa and mu opioid, sigma-1 and sigma-2, and 5-HT_2A_ and 5-HT_3_ receptors, as well as the dopamine and serotonin transporters [21]. Regarding noribogaine, its pharmacological profile is similar to the parent drug but displays some differences: it shows a higher interaction with the serotonin reuptake transporter SERT and the kappa opioid receptor. It also exhibits a lower affinity to NMDAR with no reported affinity for the 5-HT_2A_ and 5-HT_3_ receptors [22,23,24]. Although a recent study using noribogaine at low doses in humans did not observe any psychedelic effects [25], there are currently no reports studying the psychedelic properties of noribogaine at doses equivalent to those recognized for producing oneirogenic effects with ibogaine in humans. Thus, the psychedelic potential of noribogaine needs to be further explored.

In contrast to classic psychedelics, ibogaine and noribogaine present marginal or no affinity to 5-HT_2A_R, showing pharmacological similarities to other atypical psychedelics such as ketamine (NMDA-R antagonist) [26] and salvinorine A (kappa opioid receptor agonist) [27]. Nevertheless, the potential role of the 5HT_2A_R in mediating ibogaine and noribogaine effects cannot be ruled out. Previous drug discrimination studies suggested that ibogaine administration in rodents could exert some of its actions involving 5-HT_2A_R [28] In fact, activation of 5-HT_2A_R has been linked to an awaked state with features of REM sleep following systemic administration of ibogaine in rats [29,30]. In addition, noribogaine has been shown to induce structural neural plasticity in cultured embryonic rat cortical neurons, an effect probably linked to 5-HT_2A_R activation [31]. Thus, 5-HT_2A_R could be involved in the effects of these alkaloids, but indirectly and not through a direct drug–receptor interaction. 

Therefore, we decided to test the hypothesis that molecular, electrophysiological, and behavioral responses of noribogaine in mice might be dependent on the presence of 5HT_2A_R. Given the significance of sex and gender differences observed in the impact of psychiatric illnesses and the responses to pharmacological treatments [32], we also decided to study those effects of noribogaine in both female and male mice. To the best of our knowledge, information on sex differences in psychedelic effects is extremely limited. In the case of ibogaine, preclinical studies have shown sex-specific effects in rodents. For example, after ibogaine’s administration, females showed higher plasma bioavailability and concentration in the brain than male rodents [33,34]. Yet, to our knowledge, there are no previous preclinical reports studying sex differences following noribogaine administration.

In the present study we examined the role of 5-HT_2A_R on a single noribogaine dose (10 or 40 mg/kg) on 5-HT_2A_ receptor knockout (KO) (5-HT_2A_^−/−^) and wild type (WT; 5-HT_2A_^+/+^) male and female mice. We quantified locomotor activity as well as mPFC gene expression (the mRNA of immediately early genes, and glutamatergic and serotonergic receptors by qPCR), and locomotor activity following a single administration of noribogaine. We also evaluated sex-dependent effects on noribogaine-mediated changes in glutamatergic excitatory transmission (presynaptic and postsynaptic), using whole-cell patch-clamp recordings of mPFC layer V pyramidal neurons from both genotypes. 

## 2. Results

### 2.1. A Single Administration of Noribogaine Produced Differential Effects on Locomotion in Male vs. Female Mice

**Figure 1:**
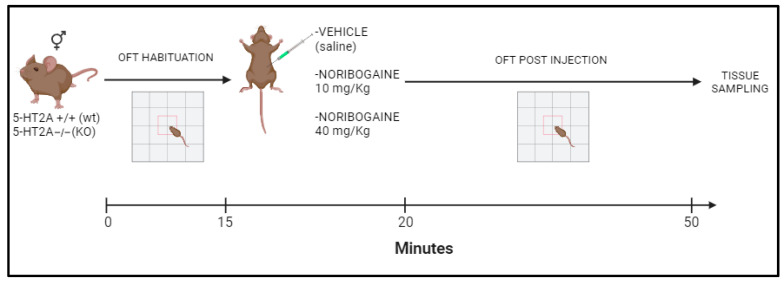
Schematic representation of experimental treatments. Male and female 5-HT_2A_^+/+^ (WT) and 5-HT_2A_^−/−^ (KO) mice were subjected to an open-field test (OFT). The first 15 min corresponds to habituation. Then, they were administered a single intraperitoneal injection of the corresponding treatment: vehicle (saline solution), noribogaine 10 mg/kg, or 40 mg/kg. Mice were recorded for 30 min post-injection. Tissue samples for qPCR were taken immediately after the experiment.

**Figure 2:**
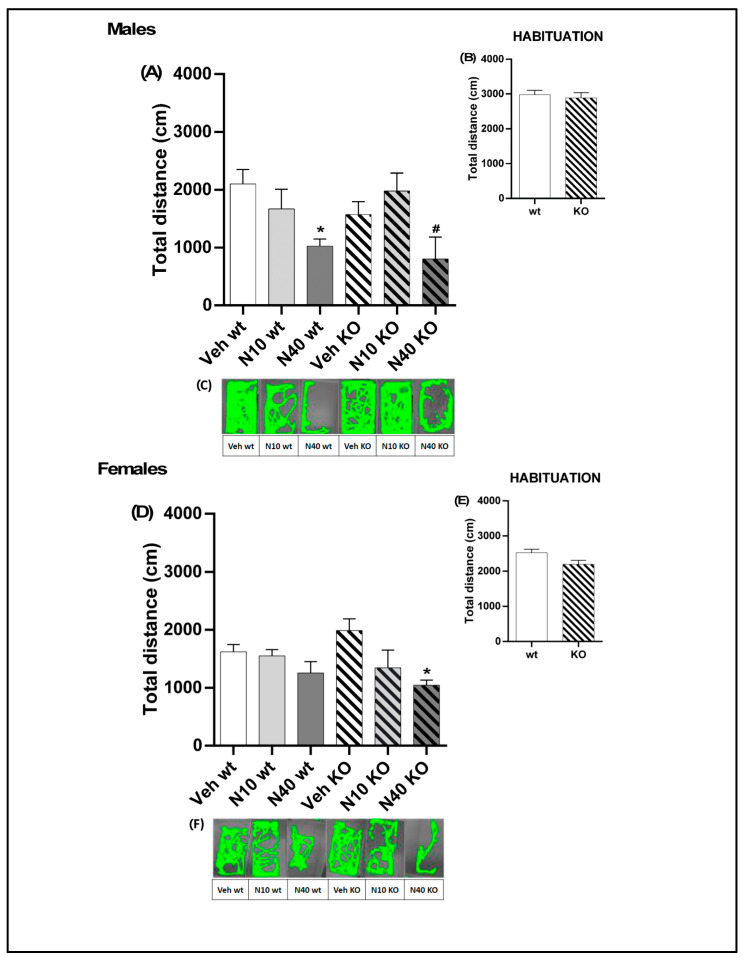
Behavioral changes induced by noribogaine (N10, 10 mg/kg or N40, 40 mg/kg). For males: (**A**) Total distance traveled (cm) by mice post-injection with noribogaine. (**B**) Total distance traveled (cm) by mice during habituation. (**C**) Cumulative track plots (screen-captured from an Ethovision file) of mice during OFT post-injection with noribogaine. For females: (**D**) Total distance traveled (cm) by mice post-injection with noribogaine. (**E**) Total distance traveled (cm) by mice during habituation. (**F**) Cumulative track plots (screen-captured from Ethovision files showing openfield arenas used for locomotion measurements, see Methods for details) of mice during OFT post-injection with noribogaine. The values indicate mean ± SEM. Two-way ANOVA–Tukey: * *p* < 0.05 different from vehicle; ^#^ *p* < 0.05 different from noribogaine (low, N10 vs. high, N40 dose).

Previous reports found that noribogaine systemic administration can change locomotor activity in rats [35]. In order to investigate whether 5-HT_2A_R deficiency would alter noribo effects on locomotion we decided to quantify locomotion of male and female 5-HT_2A_R KO (5-HT_2A_^−/−^) and WT (5-HT_2A_^+/+^) mice with a 129S6/SvEv background following the administration of psychedelic noribogaine 10 mg/kg (*Noribo 10*) or noribogaine 40 mg/kg (*Noribo 40*). First, we evaluated the basal locomotor activity in the habituation sessions prior to drug injections during 15 min (see Figure 1 for protocol details). Two-way ANOVA (sex x genotype) was performed. Locomotion in the habituation period showed a significant effect for the sex factor (F_(1,120)_ = 21.11, *p* < 0.0001, N = 26–34), but not for genotype (F_(1,120)_ = 2.72, *p* > 0.05, N = 26–34) or interaction (F_(1,120)_ = 0.90, *p* > 0.05, N = 26–34) (Figure 2B,E). According to a previous report, the max brain concentration of noribo after an intraperitoneal administration in rats occurs at 30 min [15]. Therefore, we measured locomotor activity thirty minutes after a single noribo administration, immediately after habituation. 

Two-way ANOVA (treatment x genotype) was performed. For male mice (Figure 2A–C), noribo induced significant differences for treatment (F_(2,57)_ = 8.7, *p* < 0.001, N = 9–12), but not for genotype (F_(1,57)_ = 0.52, *p* > 0.05, N = 9–12) or interaction (F_(2,57)_ = 1.5, *p* > 0.05, N = 9–12). As shown in Figure 2A, WT male mice injected with *Noribo 40* showed decreased locomotion, compared to the vehicle (Tukey post-hoc; *p* < 0.05). Female mice injected with noribo (Figure 2D–F) showed significant effects for treatment (F_(2,52)_ = 6.42, *p* < 0.001, N = 7–11), but not for genotype (F_(1,52)_ = 0.01, *p* > 0.05, N = 7–11), or interaction (F_(2,52)_ = 1.74, *p* > 0.05, N = 7–11). Figure 2D showed that female KO mice injected with *Noribo 40* showed less locomotion compared to vehicle KO (Tukey post-hoc; *p* < 0.05). 

In this experimental setting, we did not observe any significant difference for female or male mice treated with the lower dose, *Noribo 10*. Only the highest dose, *Noribo 40*, was able to change locomotion in WT male mice and in KO females. The noribo-mediated decrease in locomotion reported here might be linked to the fact that noribo is a G-protein biased k-opioid receptor agonist [36]. It is known that k-opioid agonists reduce rearing, motility, and locomotion in mice [37]; therefore, the decrease in locomotion seen in noribo-treated mice might reflect sedative effects induced by the activation of these receptors. Also, we cannot rule out the effect of noribo on the animal’s overall motivation to explore. 

These results suggest that 5HT_2A_R deficiency does not play a role in noribo locomotor effects for male mice, since both genotypes showed reduced locomotion. Nevertheless, for female mice the reduction of locomotion was observed for KO, but was not evident for WT, suggesting a differential role of 5HT_2A_R in modulating the locomotor and/or exploratory behavior in females.

### 2.2. 5-HT_2A_ Receptor Deficiency Alters the Gene Expression Profile Induced by Noribogaine in mPFC in a Sex-Dependent Manner

Cortical neurons express several 5-HT receptors, among which the 5-HT_2A_ and 5-HT_1A_ receptors are expressed at high levels [7,8,9,10,38]. 

Therefore, to evaluate the impact of 5-HT_2A_R deficiency on the mPFC gene expression profile induced by a single administration of noribo injection, we tested the same two noribo doses (*Noribo 10*, *N10* and *Noribo 40*, *N40*, see above). Male and female mice were injected with noribo or vehicle, tested in the open field for locomotion activity (for 30 min), and tissue sampling (mPFC) was immediately obtained for qPCR assays (IEGs, glutamate receptors, and 5-HT_2A_ receptors) (see Figure 1 for protocol details).

**Figure 3:**
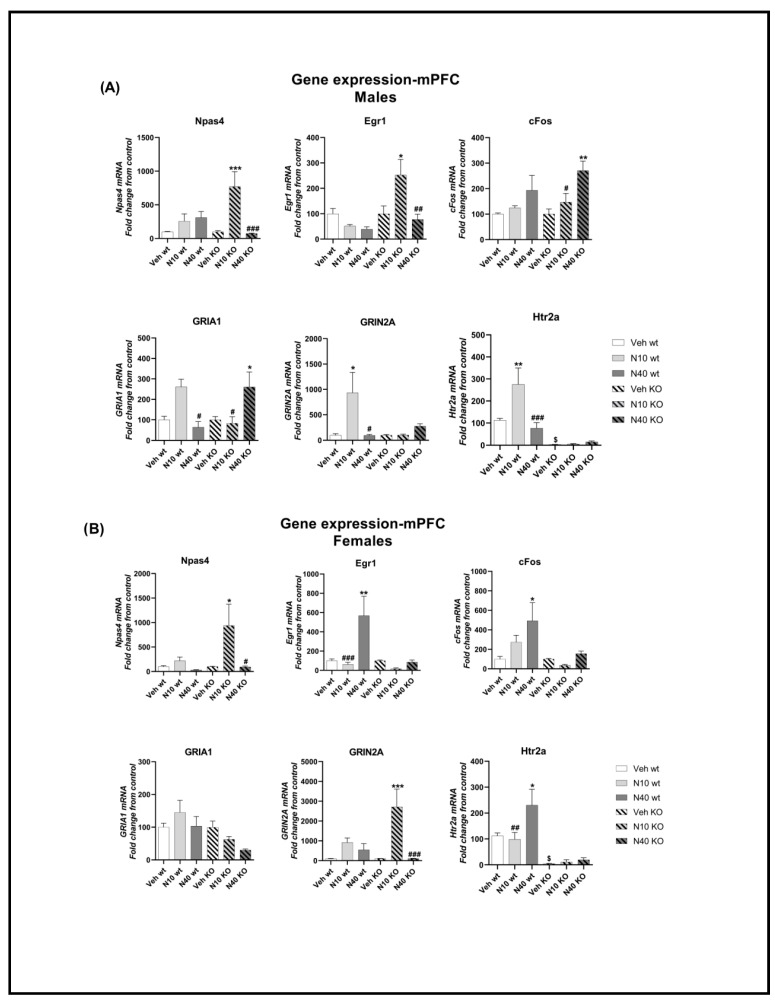
Gene expression changes in WT and KO mice in the mPFC following a single injection of noribogaine (10 mg/kg or 40 mg/kg). (**A**) Males: Immediate early genes Npas4, Egr1, and cFos; glutamate receptor GRIA1 and GRIN2A; serotonin receptor Htr2a (5HT_2A_R). Two-way ANOVA–Tukey: * *p* < 0.05, ** *p* < 0.01, *** *p* < 0.001 difference from vehicle (KO injected with noribo vs. vehicle KO and WT injected with noribo vs. vehicle WT); # *p* < 0.05, ## *p* < 0.01, ### *p* < 0.001 difference from noribogaine (comparing low vs. high dose); $ *p* < 0.05 difference from vehicle (vehicle WT vs. vehicle KO). (**B**) Females: Immediate early genes Npas4, Egr1, and cFos; glutamate receptor GRIA1 and GRIN2A; serotonin receptor Htr2a. Two-way ANOVA–Tukey: * *p* < 0.05, ** *p* < 0.01, *** *p* < 0.001 difference from vehicle (KO injected with noribo vs. vehicle KO and WT injected with noribo vs. vehicle WT); # *p* < 0.05, ## *p* < 0.01, ### *p* < 0.001 difference from noribogaine (comparing low vs. high dose); $ *p* < 0.05 difference from vehicle (vehicle WT vs. vehicle KO).

For male mPFC (Figure 3A), Npas4 showed a significant main effect of treatment (F_(2,48)_ = 9.00, *p* < 0.001, N = 7–11) and interaction (F_(2,48)_ = 6.32, *p* < 0.01, N = 7–11), but not for genotype (F_(1,48)_ = 1.13, *p* > 0.05, N = 7–11). As shown in Figure 3, male KO mice injected with *Noribo 10* showed higher Npas4 expression compared to vehicle KO (Tukey post-hoc, *p* < 0.001). Egr1 showed significant treatment (F_(2,46)_ = 3.41, *p* < 0.05, N = 7–10), genotype (F_(1,46)_ = 7.66, *p* < 0.01, N = 7–10), and interaction (F_(2,46)_ = 4.82, *p* < 0.05, N = 7–10). Similarly to what we observed for Npas4, administration of *Noribo 10* in male KO mice increased Egr1 expression compared to vehicle KO (Tukey post-hoc, *p* < 0.05). For cFos, ANOVA showed differences for treatment (F_(2,49)_ = 9.97, *p* < 0.001, N = 7–11) but not genotype (F_(1,49)_ = 1.76, *p* > 0.05, N = 7–11) nor interaction (F_(2,49)_ = 0.84, *p* > 0.05, N = 7–11). cFos expression was increased in KO male mice injected with the higher *Noribo 40* dose compared to vehicle KO (Tukey post-hoc, *p* < 0.01). We did not observe significant differences in WT mice on IEGs expression following noribo administration.

We also measured the mRNA of two glutamate receptors that are linked to neuroplasticity-related changes in mPFC circuits in male mice (Figure 3A). The AMPA receptor GRIA1 showed significant effects for interaction (F_(2,50)_ = 9.82, *p* < 0.001, N = 6–11), but not for treatment (F_(2,50)_ = 2.02, *p* > 0.05, N = 6–11) or genotype (F_(1,50)_ = 0.04, *p* > 0.05, N = 6–11). Male KO mice injected with *Noribo 40* showed increased GRIA1 compared to vehicle KO (Tukey post-hoc, *p* < 0.05). The NMDA receptor GRIN2A showed significant interaction (F_(2,45)_ = 4.188, *p* < 0.05, N = 6–10), but not for treatment (F_(2,45)_ = 3.035, *p* > 0.05, N = 6–10) nor genotype (F_(1,45)_ = 2.123, *p* > 0.05, N = 6–10). WT males injected with *Noribo 10* showed an increase in GRIN2A compared to vehicle WT (Tukey post-hoc, *p* < 0.05). 

As expected, male KO showed a decrease in the 5-HT_2A_R gene compared to vehicle WT (Tukey pot-hoc, *p* < 0.05), like in a previous report using this knockout mouse model [39]. 5-HT_2A_R showed statistical differences for all factors, including treatment (F_(2,47)_ = 7.40, *p* < 0.01, N = 7–10), genotype (F_(1,47)_ = 47.95, *p* < 0.0001, N = 7–10), and interaction (F_(2,47)_ = 8.27, *p* < 0.001, N = 7–10). Interestingly, the lower dose, *Noribo 10*, induced an increase in 5-HT_2A_R gene expression in WT male mice compared to vehicle WT (Tukey post-hoc, *p* < 0.01). Our results suggest that noribo has the capacity to regulate the expression of 5-HT_2A_R mRNA.

For the female mPFC (Figure 3B), we noted significant changes for Npas4 that were similar to males for treatment (F_(2,42)_ = 5.04, *p* < 0.05, N = 6–10), but not for genotype (F_(1,42)_ = 3.05, *p* > 0.05, N = 6–10) nor interaction (F_(2,42)_ = 2.45, *p* > 0.05, N = 6–10). Like male mice, female KO injected with *Noribo 10* increased Npas4 in mPFC compared to vehicle KO (Tukey post-hoc, *p* < 0.05). In the case of Egr1, significant differences were found for treatment (F_(2,45)_ = 6.81, *p* < 0.01, N = 7–9), genotype (F_(1,45)_ = 6.73, *p* < 0.05, N = 7–9), and interaction (F_(2,45)_ = 5.28, *p* < 0.01, N = 7–9). WT female mice injected with *Noribo 40* increased its expression compared to vehicle WT (Tukey post-hoc, *p* < 0.01). Also, cFos showed differences for treatment (F_(2,45)_ = 4.77, *p* < 0.05, N = 7–10) and genotype (F_(1,45)_ = 9.89, *p* < 0.01, N = 7–10), but not for interaction (F_(2,45)_ = 2.75, *p* > 0.05, N = 7–10). Female WT injected with *Noribo 40* increased cFos expression compared to vehicle WT (Tukey pot-hoc, *p* < 0.05).

GRIA1 showed statistical differences for genotype (F_(1,42)_ = 6.89, *p* < 0.05, N = 7–10), but not for treatment (F_(2,42)_ = 1.36, *p* > 0.05, N = 7–10) nor interaction (F_(2,42)_ = 1.83, *p* > 0.05, N = 7–10). No other significant differences were found (Tukey post-hoc, *p* > 0.05). GRIN2A showed differences for treatment (F_(2,41)_ = 11.23, *p* < 0.01, N = 6–10) and interaction (F_(2,41)_ = 4.4, *p* < 0.05, N = 6–10), but not for genotype (F_(1,41)_ = 1.81, *p* > 0.05, N = 6–10). KO female mice injected with *Noribo 10* showed increased GRIN2A expression compared to vehicle KO (Tukey post-hoc, *p* < 0.001).

Similarly to male mice, we found significant differences in the expression of 5HT_2A_R, for treatment (F_(2,45)_ = 4.55, *p* < 0.05, N = 6–10) and genotype (F_(1,45)_ = 41.68, *p* < 0.0001, N = 6–10) but not for interaction (F_(2,45)_ = 3.16, *p* > 0.05, N = 6–10). WT female mice injected with *Noribo 40* showed increased 5-HT_2A_R expression compared to vehicle WT (Tukey post-hoc, *p* < 0.05). As expected, KO female mice showed decreased expression of 5HT_2A_R expression compared to female vehicle WT (Tukey post-hoc, *p* < 0.05).

### 2.3. 5-HT_2A_ Receptor Plays a Role in the NMDA Current Density of Pyramidal mPFC Neurons in Male WT Mice Following Single Administration of Noribogaine

**Figure 4:**
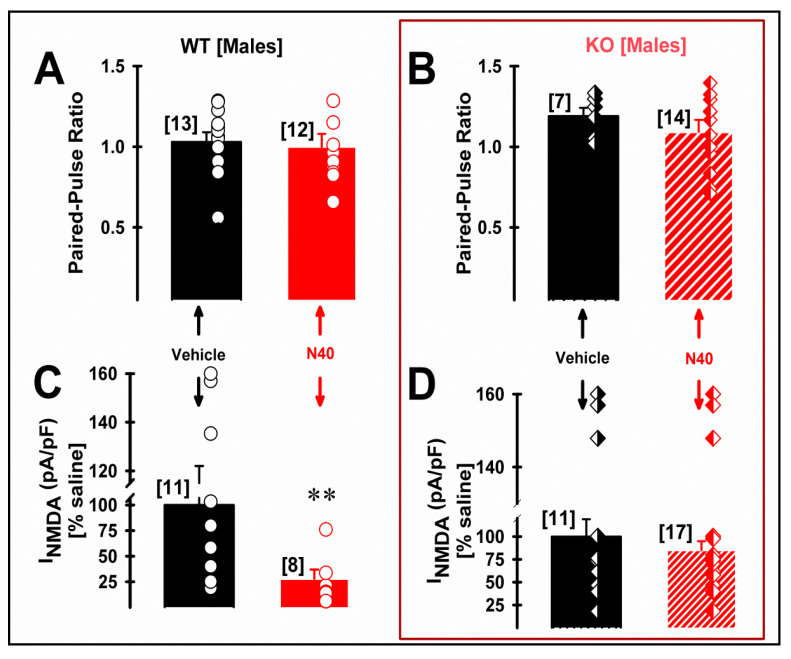
Systemic noribogaine (*Noribo 40*; N40) treatment reduced NMDA-mediated current density in mPFC pyramidal neurons from male mice. (**A**,**B**) Graphs showing saline (black bars) and N40 (red bars) administration effect on mean EPSC paired-pulse ratio during 10 Hz stimulation (i.e., fraction of EPSC_2_/EPSC_1_ amplitudes) recorded from layer V mPFC pyramids from male WT (left plot) and 5HT_2A_ KO (right plot) mice, respectively. Individual paired-pulse ratio values are shown for each treatment as overlying (WT), rhombi (5HT_2A_ KO) on each bar. No significant differences were observed comparing PPR values. (**C**,**D**) Graphs showing saline (black) and N40 (red bars) administration effects on mean NMDA-mediated current density (pA/pF) values from male WT (left plot) and 5HT_2A_ KO (right plot) mice. Individual NMDA-mediated current density values are shown for each treatment as overlying circles (WT), rhombi (5HT_2A_ KO) on each bar. Note how N40 was able to significantly reduce the NMDA current density only in pyramidal neurons from WT male mice (see Table 1 for statistical comparisons). ** Mean INMDA current-density values were significantly smaller in KO compared to WT (post-hoc Tukey test; q = 6.2, *p* < 0.01). The values indicate mean ± SEM. The number of cells recorded for each group were included in brackets on top of each bar.

Postsynaptic membrane expression of NMDA receptors in dendrites of layer V pyramidal neurons of mPFC has been described to be finely tuned by the activation of 5-HT_2A_R activation [38]. Therefore, we studied the effects of systemic *Noribo 40* and genotype (both sexes WT vs. 5HT_2A_ KO mice) on presynaptic (paired-pulse ratio) and postsynaptic NMDA current density during whole-cell patch clamp recordings of layer V pyramidal cells (see Supplementary Figure S1 for methodological details). Figure 4 summarize Noribo 40-mediated (saline vs. N40) changes at a presynaptic (i.e., paired-pulse ratio) and postsynaptic (i.e., NMDA current density; in pA/pF) of layer V pyramidal neurons in mPFC coronal slices from male mice of both genotypes. 

Presynaptic changes in the probability of excitatory synaptic transmission were studied using paired-pulse ratios during 10 Hz stimulation, dividing the amplitude of a second post-synaptic response by that of the first [40]. In males, Figure 4A,B show no presynaptic changes after *Noribo 40* treatment, as observed by the absence of significant differences of mean paired-pulse ratios for either genotype of male mice. Figure 4C,D show a significant reduction in postsynaptic NMDA-mediated current density from male WT mice treated with *Noribo 40*, but not in KO mice. 

Table 1 shows statistical comparisons among all synaptic results from male mice, highlighting that significantly smaller NMDA-mediated current density values were observed in pyramidal neurons from KO male mice compared to WT. As expected, the NMDA/AMPA ratio [41] was also significantly smaller after *Noribo 40* treatment in male WT. Interestingly, KO male mice showed similar low NMDA/AMPA ratios (Table 1).

**Figure 5:**
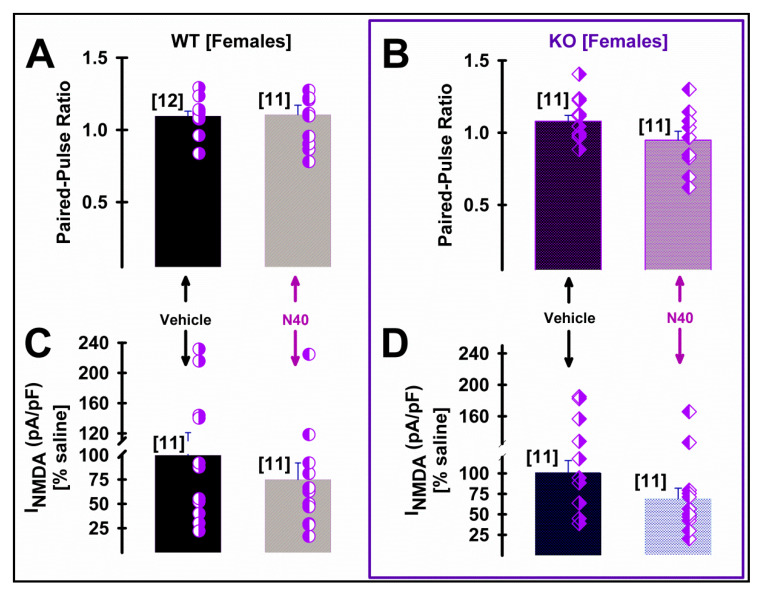
Systemic noribogaine (*Noribo 40*; N40) did not alter synaptic parameters of mPFC pyramidal neurons from female mice. (**A**,**B**) Graphs showing the effects of saline (black bars) and N40 (grey bars) administration on the mean EPSC paired-pulse ratio during 10 Hz stimulation (i.e., fraction of EPSC2/EPSC1 amplitudes) recorded from layer V mPFC pyramid neurons from female WT (left plot) and 5HT_2A_ KO (right plot) mice. Individual paired-pulse ratio values are shown for each treatment as overlying (WT), rhombi (5HT_2A_ KO) on each bar. The number of cells included in each bar is shown in brackets. (**C**,**D**) Graphs showing the effects of saline (black bars) and noribogaine (grey bars) administration on mean NMDA-mediated current density (pA/pF) values from female WT (left plot) and 5HT_2A_ KO (right plot) mice. Individual I_NMDA_-density pulse values are shown on each bar for each treatment (see overlying circles for WT, and rhombi for 5HT_2A_ KO). The number of cells included in each bar is shown in brackets. No significant differences were observed comparing PPR values (see Table 2 for statistical comparisons). The values indicate mean ± SEM. The number of cells recorded for each group were included in brackets on top of each bar.

On the other hand, Figure 5 shows no effect of *Noribo 40* on either presynaptic or postsynaptic parameters measured in females form either WT or KO mice. Table 2 details statistical comparisons among female groups, showing no significant differences. 

## 3. Discussion

The mPFC and other cortical brain regions are involved in functions such as emotional regulation, cognitive processing, and introspection, and it has been shown that psychedelics can influence the activity in specific “hub” cortical regions of the brain, affecting the ability to coordinate neural activity in downstream brain regions [42]. High psychedelic doses induce vivid perceptual experiences that are associated with a therapeutic benefit, but there have also been anecdotal reports of these drugs being used in a lower dose to improve cognitive functions. Recently, in a preclinical model, Higgins et al. [43] reported that low doses of psilocybin and ketamine enhance motivation and attention in poor-performing rats. These data suggest that even low doses of psychedelics might improve symptoms that rely on mPFC functions. 

Immediate early genes (IEG) can become highly expressed within seconds or minutes of endogenous or exogenous stimuli like cFos [44]. IEG have also been linked to learning such as for Egr-1 (early growth response gene; a.k.a. zif268) [45] or synaptic plasticity for Npas4 (neuronal Per-Arnt-Sim domain protein 4) [46]. We found that in mPFC, noribo induced a 5-HT_2A_R-dependent regulation of gene expression on several IEGs (Npas4, Egr1, cFos), in a sex-dependent manner. 

It needs to be noted that to the best of our knowledge this is the first study investigating the effects of psychedelics on Npas4 expression, showing that noribo can increase Npas4 expression mRNA in mPFC. This IEG is expressed throughout the brain at a low level, though it is enriched in the frontal, parietal, and entorhinal cortices [46,47]. The primary signal for inducing Npas4 is an increase in intracellular calcium (Ca^2+^) concentration that, in neurons, is regulated by excitatory neurotransmission [46]. Thus, Npas4 is thought to regulate the balance of excitatory/inhibitory neurotransmission within cortical loops.

Serotonergic neurotransmission and, more particularly, activation of 5-HT_2A_-Rs in the PFC play a critical role in the regulation of cortical circuits [48]. 5-HT_2A_R is extensively distributed, particularly in medial frontal cortex, where postsynaptic activation modulates pyramidal glutamatergic neuron activity and participates in various executive functions [49]. Depressive patients commonly suffer from cognitive dysfunction comprising working memory, problem solving, and cognitive flexibility [50]. It has been shown that genetic loss of the 5-HT_2A_R compromised the activity of chronic treatment with SSRI (selective serotonin reuptake inhibitors), making this receptor a putative marker predicting antidepressant responses [51].

Beyond the previously described NMDA antagonist mechanism for ibogaine [52,53], we took advantage of an available genetically modified mouse model for the 5-HT_2A_ receptor [9,10] in order to characterize the potential contribution of this receptor to postsynaptic NMDA current density following Noribo 40 administration. In male mice, electrophysiological results showed a reduction of NMDA-mediated current density only in WT mice. Similarly, NMDA/AMPA ratios [41] were reduced after Noibo 40 administration only in WT male mice. No changes in presynaptic paired-pulse ratios were observed in any experimental conditions, regardless of the described expression of 5-HT_2A_R at presynaptic glutamatergic afferents [7]. Our electrophysiology patch-clamp results further suggest the existence of a functional link between postsynaptic 5HT_2A_R and NMDA that can be altered by the genetic removal of 5-HT_2A_ receptors in male mice. Since noribo does not interact directly with this receptor, one possible explanation could be related to its effects as a SERT inhibitor, increasing serotonin availability that in turn binds to all serotonin receptors. For some of the effects described in this study, it is not farfetched to expect that lacking 5-HT_2A_R would interfere with noribo pharmacological responses. 

A previous report has described that 5-HT_2A_ receptor activation might counteract the inhibiting effect of 5-HT_1A_ receptor activation on postsynaptic NMDA receptors’ membrane expression [38]. 5-HT_2A_ receptors were shown to activate ERK via β-arrestin-dependent pathways, stabilizing dendritic microtubules and NMDA receptors’ membrane expression [38]. In agreement with that, our results show less NMDA current density in pyramidal neurons recorded in mPFC slices after systemic treatment with Noribo 40 treatment in WT as wells as from KO, but only in male mice. Yuen et al. [38] used cell cultures and prefrontal cortex slices from male rats. Our results show no synaptic changes in layer V pyramidal neurons from female mice, suggesting a sex-dependent difference in noribo-mediated NMDA-blocking properties (both WT and KO). Considering previous reports [33], the present results observed in female mice should not be due to sex-dependent changes in brain or plasma noribo levels. 

### The Neuroprotective and Neuroplastic Potential of Noribogaine at mPFC Pyramidal Neurons: The Contribution of NMDA and 5-HT_2A_ Receptors to Rapidly Promoting Plasticity

Neuroprotection and neuroplasticity can be viewed as continuous adaptations of the neurons to new functional scenarios involving different mechanisms directed against harmful elements. Neuroplasticity involves the active restoration of a new biological baseline following environmental or pharmacological challenges.

Several psychoactive drugs with clinical use regulate the expression of neurotrophic factors, a process called induced plasticity (iPlasticity), which allows network reorganization in the adult brain [54]. This is in accordance with this fact; noribo has been recently classified as a “psychoplastogen” since it can promote rapid and maintained neuritogenesis in cultured rat cortical neurons [31]. In this manner, neuroplastic changes driven by several synaptic modulations might underlie behavioral changes induced by psychedelics. For instance, it is well established that an increase in serotoninergic neurotransmission leads to an increase in BDNF expression/signaling both in vitro and in vivo [55,56]. Thus, sustained enhancement of serotonin transmission due to ibogaine and its long-lasting metabolite noribogaine could account, at least in part, for the observed effect on BDNF and GDNF expression after ibogaine administration [57].

Npas4 plays an important role in protecting neurons against many types of neurodegenerative insults. NPAS4 is selectively expressed in neurons following membrane depolarization-induced intracellular calcium signaling. Pollina et al. [58] proposed that the formation of a complex between NPAS4 and NuA4 may represent a mechanism by which neurons efficiently drive activity-induced transcriptional responses while simultaneously preserving genome stability [58]. Sustaining neuronal “vitality” over time appears to require careful balancing of the proper ratio of excitation and inhibition. The present study shows that Npas4 expression is increased in KO female and male mice post noribo administration, suggesting that Npas4 regulation by noribo needs low levels of 5-HT_2A_R expression and NMDA-density to occur. This points out that in a context of low neurotransmission at the 5-HT_2A_R that may favor the susceptibility to major depressive disorder [59], noribo might in turn facilitate the expression of this neuroprotective IEG and contribute to its profile as a potent antidepressive drug.

In postmortem tissue from patients suffering from major depressive disorder, Egr1 levels in the prefrontal cortex are lower when compared to healthy controls [60]. Notably, such a reduction was observed in both unmedicated and medicated subjects not responding to treatment and thus suggests that EGR1 levels in the mPFC are directly associated with a depressive phenotype and could be seen as a marker or mediator of a positive response to antidepressant treatment [60]. In light of the tight link between EGR1 expression and neuronal plasticity, the downregulation of EGR1 in the mPFC of depressed patients is particularly interesting and could represent one of the substrates for the anatomical and functional alterations observed in major depressive disorders in this brain area [61,62]. Therefore, our results showing that noribo can increase the expression of Egr1 in KO male mice and WT female mice can be interpreted as noribo increasing neuroplastic effectors like Egr1 (in a sex-dependent manner). Low levels of Egr1 expression are linked to depression, but they can also serve as a marker for positive pharmacological treatment outcomes when its expression is increased or restored. 

One of the new approaches to the treatment of depression is focused on glutamatergic neurotransmission. It has been shown that a blockade of the NMDA receptor complex creates new opportunities for the treatment of affective disorders [63]. The NR2B subunit selective NMDA antagonist, traxoprodil, co-administered with agents that affect monoaminergic neurotransmission (like SSRI) at inactive doses, produced a significant antidepressant-like effect in the forced swim test in mice [64]. NMDARs are activated in response to neuronal depolarization and Ca^2+^ entry; thus, NMDA antagonism could mediate neuroprotection. It would reduce the amount of Ca^2+^ entry at distal dendrites of layer V pyramidal cells. In cortical networks, NMDA blockage would reduce plastic long-term synaptic events during high-frequency stimulation. Therefore, it can be suggested that some of the beneficial effects of noribo on depression might be associated with a reduction of NMDA receptor activation leading to neuroprotective effects and neuroplastic changes in mPFC networks. This NMDA effect on mPFC induced by noribo seems to be sex-dependent; thus, it might be suggested that female and male subjects may differentially experience noribo’s beneficial effects in clinical settings. Our results support the importance of studying sex as a biological variable in preclinical psychedelic research. It needs to be mentioned that when using psychedelics, not only the gonadal axis might be responsible for gender differences, but also the stress response along the HPA axis might play a role. We already know from research outside of psychedelics, that these two axes do impact each other: stress responses can impact sexual hormones and vice versa. Also, we cannot completely rule out that sex differences in noribo pharmacokinetics may also play a role in some of the variables investigated in our study.

## 4. Material and Methods

### 4.1. Animals

The 5-HT_2A_ receptor knockout mouse line was kindly provided by Dr. Noelia Weisstaub (Universidad Favaloro, Buenos Aires, Argentina). The generation of genetically modified *5-HT_2A_^−/−^* mice and their control (WT or *5-HT_2A_^+/+^*) littermates was described elsewhere [9], and genotypes were confirmed using PCR analysis (see the Supplemental Information for primer sequences). Males and females (8–12-week-old) were housed in a light- and temperature-controlled room throughout the experimental procedures with water and food ad libitum, under a 12 h light/dark cycle (lights on at 8:00 a.m.) at a temperature of 21–23 °C. All efforts were made to minimize animal suffering and to reduce the number of animals used. All animal experiments were conducted in accordance with institutional guidelines in compliance with national and international laws and policies (‘Guidelines for the Care and Use of Mammals in Neuroscience and Behavioral Research’, National Research Council, 2003, and OLAW and ARENA directives, NIH, Bethesda, USA).

All experiments were approved by the Institutional Animal Care and Use Committees of Universidad de Buenos Aires, UBA, CICUAL-FCEN-UBA #169, 2022–2025 and Universidad Austral, CICUAL-IIMT, 2023-04, 2023–2026.

### 4.2. Drugs

Noribogaine-HCl was prepared in the Laboratorio de Síntesis Orgánica-Facultad de Química—Universidad de la República (Montevideo, Uruguay) using ibogaine as a starting material. Ibogaine was obtained as described in our previously reported method [12] via the decarboxylation of voacangine (See Supplementary Figures S2 and S3). In brief, the experimental procedure to obtain noribogaine from ibogaine was as follows: in a two-neck round-bottom flask under an argon atmosphere, ibogaine (493 mg, 1.58 mmol) was dissolved in dry 1,2-dichloroethane (15.8 mL, 0.1 M) using magnetic stirring. Then, ethanetiol (497 µL, 6.34 mmol, 4.0 eq) was added followed by the addition of a 1.0 M solution of boron tribromide (2.38 mL, 1.5 eq). The system was heated to 55 °C for 2 h, when total consumption of the starting material was evidenced by thin-layer chromatography (TLC). The reaction was quenched by adding methanol until all the suspended solids were dissolved. The resulting reaction mixture was diluted with EtOAc and transferred to a separation funnel, where a saturated sodium bicarbonate solution was added. The aqueous layer was extracted with ethyl acetate (EtOAc x3), and the combined organic layers were dried over sodium sulfate. The solvent was removed under vacuum to obtain a crude mixture that was purified using column chromatography (SiO_2_; 50% EtOAc in Hexane, 1% NH_4_OH(cc)). The noribogaine free base was obtained as a white amorphous solid (420 mg, 89% yield). Structural characterization of this material as pure noribogaine was assessed via nuclear magnetic resonance (1H, COSY, 13C, HSQC and HMBC; see the Supporting Information). To prepare the corresponding hydrochloride, the free base was dissolved in dry diethyl ether and an anhydrous solution of HCl in diethyl ether (3 M, 0.75 mL, 1.5 eq) was added. A white solid was formed, which was filtrated and washed several times with dry diethyl ether. The resulting noribogaine-HCl (425 mg) was dried under vacuum and obtained as a white solid. Dissolution of noribogaine-HCl to prepare the samples for i.p. injection was carried out using a warm saline vehicle that was previously degassed via nitrogen bubbling.

### 4.3. Behavioral Test

#### Locomotor Activity

Mice locomotor activity (total distance, in cm) was recorded using a CCD camera (Sony, New York, NY, USA) on custom-designed open-field boxes located in a sound-attenuated room. For acquisition and analysis, we used Ethovision XT 11.0 software (Noldus, Wageningen, The Netherlands). Each box consisted of an open-field plastic compartment (19 cm × 40 cm × 40 cm). Animals were placed in open-field boxes for 15 min (recorded as baseline) and later injected with drugs or saline. Total distance traveled (in cm) was quantified for a total of 30 min after injections. Behavioral recordings were made simultaneously in four open-field arenas using Ethovison XT 5.1 multiple-arena features from 9 a.m. to 4 p.m. of the light period of the photocycle (like in a previously published study, see [65]). Injection time and arenas (right and left) were fully counterbalanced among subjects and experimental groups.

### 4.4. Real-Time qPCR

Prefrontal cortex tissue was extracted immediately after behavioral testing: mouse brains were rapidly removed; striatal tissues were dissected, placed on dry ice, and then stored at −70 °C until further assays. Total RNA was isolated using TRIZOL reagent (Invitrogen, Carlsbad, CA, USA) according to the manufacturer’s protocol. Five hundred nanograms of RNA were treated with DNAseI (Invitrogen) and reverse-transcribed in a 20 µL reaction using M-MLV reverse transcriptase (Promega, Madison, WI, USA) and random hexameres (Biodynamics, Ciudad de Buenos Aires, Argentina). For quantitative real-time PCR (qPCR), primers sets were designed for the specific amplification of murine genes and actin-B as a housekeeping control gene (sequences listed in Table 3). Each sample was assayed in duplicate using 4 pmol of each primer, 1X SYBR Green Master Mix (Applied Biosystems, Foster City, CA, USA) and 2–20 ng of cDNA in a total volume of 13 µL. Amplification was carried out in an ABI PRISM 7500 sequence detection system (Applied Biosystems). See Table 3 for the primers sequence. 

### 4.5. Single-Cell Electrophysiological Recordings in Slices

The researcher in charge of electrophysiological recordings, analysis of data, and statistical comparisons was blind to the mice genotype. Half an hour after saline or noribogaine (40 mg/kg, i.p.), mice were deeply anesthetized (ketamine and xylazine) followed by decapitation. A group of 16 WT (11 males and 5 females) and 17 KO (11 males and 6 females) of 4–8-week-old mice was used for whole-cell patch clamp recordings. Coronal brain slices, including mPFC (300 μm), were obtained by gluing both hemispheres with the caudal part onto a vibratome stage (PELCO, EasiSlicer, Ted Pella Inc., Redding, CA, USA), and submerged in a chamber containing chilled low-sodium/high-sucrose solution (composition in mM: 250 sucrose, 2.5 KCl, 3 MgCl_2_, 0.1 CaCl_2_, 1.25 NaH_2_PO_4_, 0.4 ascorbic acid, 3 myo-inositol, 2 pyruvic acid, 25 D-glucose, and 25 NaHCO_3_). Slices were cut sequentially and transferred to an incubation chamber at 35 °C for 30 min containing a stimulant-free, low Ca^2+^/high Mg^2+^ normal artificial cerebrospinal fluid (ACSF) (composition in mM: 125 NaCl, 2.5 KCl, 3 MgCl_2_, 0.1 CaCl_2_, 1.25 NaH_2_PO_4_, 0.4 ascorbic acid, 3 myo-inositol, 2 pyruvic acid, 25 d-glucose, and 25 NaHCO_3_ and aerated with 95% O_2_ + 5% CO_2_, pH 7.4). After incubation, slices were allowed to return to room temperature.

Whole-cell patch-clamp recordings were performed in medial prefrontal coronal slices, as previously described [40]. Recordings were made at physiological temperature (36–37 °C). Patch electrodes were made from borosilicate glass (2–3 MΩ) filled with a voltage-clamp high CsCl solution (composition in mM: 110 CsCl, 40 HEPES, 10 TEA-Cl, 12 Na_2_phosphocreatine, 0.5 EGTA, 2 Mg-ATP, 0.5 Li-GTP, and 1 MgCl_2_. pH was adjusted to 7.3 with CsOH). To block Na^+^ currents and avoid postsynaptic action potentials, 10 mM N-(2,6-diethylphenylcarbamoylmethyl) triethylammonium chloride (QX-314) was added to the pipette solution. Signals were recorded using a MultiClamp 700 amplifier commanded by pCLAMP 10.0 software (Molecular Devices, San Jose, CA, USA). Cells with series resistance (Rs) <  15 MΩ were used after being compensated online (>80%). Data were filtered at 5 kHz, digitized, and stored for off-line analysis. Visually identified pyramidal layer V neurons of the prelimbic cortex were used in this study [40]. Pyramids were visualized using Nomarski contrast on an upright BX-50WI microscope (Olympus America Inc., Center Valley, PA, USA) (40×, 0.8 numerical water-immersion objective) using a near-IR light coupled to an IR-sensitive charge-coupled device camera (DMK 23UP1300; Imaging Source, Bremen, Germany).

Evoked excitatory postsynaptic currents (EPSC) were evoked extracellularly (twice the threshold; 40–200 μs; 200–1000 μA) using a bipolar concentric electrode (FHC Inc., Bowdoin, ME, USA) located on the deep-layers border of the mPFC. Using a high CsCl/Qx314-intracellular solution described above and an extracellular ACSF solution containing MgCl_2_ (1 mM), CaCl_2_ (1 mM) and bicuculline (5–10 μM), eight to twelve stimuli of a 10 Hz paired-pulse protocol were delivered. The EPSC paired-pulse ratio was calculated as the fraction of 2nd EPSC/1st EPSC amplitudes while pyramidal cells were held at −70 mV holding. NMDA/AMPA were calculated at 50 mV holding as previously described [41]. Average current density was calculated by dividing the mean amplitude by the cell’s capacitance values obtained from MultiClamp compensation values.

### 4.6. Statistical Analysis

Data are expressed as the mean ± SEM. Statistics were performed using two-way ANOVAs followed by Tukey post-hoc multiple comparisons tests. Calculations were carried out using GraphPad Prism 7 software. All data are presented as mean ± SEM, and significance was determined at *p* < 0.05.

## 5. Conclusions

In the present study we demonstrated, for the first time, that noribo administration induced changes in the levels of transcripts of several IEGs and receptors within the mPFC in a sex-dependent manner. In addition, in some cases, this effect was found to involve 5-HT_2A_R. Differential effects on gene expression and locomotor activity were observed when comparing 10 mg/kg versus 40 mg/kg systemic administration in female and male mice. IEGs, particularly those known to be closely linked to neuroplasticity (i.e., Npas4 and Egr1), were responsive to different doses of noribo and sex. Also, our results show that a single administration of noribo has the ability of increase 5-HT_2A_R expression in mPFC and this effect might be related to its antidepressive-like properties found in pre-clinical studies.

Additionally, we showed noribo-mediated changes in NMDA synaptic transmission in the mPFC. Using the high noribo dose of 40 mg/kg, electrophysiological recordings showed selective postsynaptic effects on mPFC NMDA-mediated currents in the presence of 5-HT_2A_R (i.e., WT) of male mice, suggesting a potential functional link between postsynaptic 5HT_2A_ receptors and NMDA that can be altered by the genetic removal of 5-HT_2A_ receptors in male mice. Noribogaine did not affect presynaptic paired-pulse plasticity for glutamate release. Strikingly, no synaptic effects were observed in female mice.

## Figures and Tables

**Table 1:** Effects of systemic noribogaine administration (*Noribo 40*) on paired-pulse ratio and NMDA current density in mPFC pyramidal cells from male wildtype and 5HT_2A_ knockout (KO) mice.

	Paired-Pulse Ratio (PPR)		I_NMDA_ (pA/pF)		NMDA/AMPA Ratio	
	Wildtype	5HT_2A_ Knockout	Wildtype	5HT_2A_ Knockout	Wildtype	5HT_2A_ Knockout
Vehicle	1.03 ± 0.06 (13)	1.19 ± 0.05 (7)	6.02 ± 1.37 (11)	2.35 ± 0.45 (11) **	0.75 ± 0.04 (10)	0.55 ± 0.04 (10) ^&&^
Noribo 40	1.00 ± 0.08 (12)	1.09 ± 0.07 (14)	1.48 ± 0.56 (8) ^$^	1.95 ± 0.22 (17)	0.56 ± 0.03 (8) ^#^	0.60 ± 0.04 (10)

No significant differences were observed comparing PPR values; one-way ANOVA, F_(3,40)_ = 0.9, *p* = 0.4. One-way ANOVA showed significantly different NMDA current densities: F_(3,42)_ = 7.6, *p* < 0.001. ^$^ Mean I_NMDA_ current-density values in WT were significantly reduced by *Noribo 40* (post-hoc Tukey test; q = 6.2, *p* < 0.01). ** Mean I_NMDA_ current-density values were significantly smaller in KO compared to WT (post-hoc Tukey test; q = 6.2, *p* < 0.01). ^#^ NMDA/AMPA ratios in WT were significantly reduced by *Noribo 40*, according to one-way ANOVA F_(1,17)_ = 12.9, *p* = 0.002; post-hoc Tukey test; q = 5.1, *p* = 0.003. ^&&^ NMDA/AMPA ratios in WT were significantly larger compared to KO; one-way ANOVA F_(1,19)_ = 11.9, *p* = 0.003.

**Table 2:** Effects of systemic noribogaine administration (*Noribo 40*) on paired-pulse ratio and NMDA current density in mPFC pyramidal cells from female wildtype and 5HT_2A_ knockout (KO) mice.

	Paired-Pulse Ratio (PPR)		I_NMDA_ (pA/pF)		NMDA/AMPA Ratio	
	Wildtype	5HT_2A_ Knockout	Wildtype	5HT_2A_ Knockout	Wildtype	5HT_2A_ Knockout
Vehicle	1.09 ± 0.03 (12)	1.09 ± 0.05 (11)	2.20 ± 0.49 (11)	2.90 ± 0.45 (11)	0.49 ± 0.04 (7)	0.62 ± 0.05 (10)
Noribo 40	1.10 ± 0.07 (11)	0.95 ± 0.06 (11)	1.59 ± 0.38 (11)	1.87 ± 0.35 (11)	0.51 ± 0.05 (8)	0.60 ± 0.11 (7)

No significant differences were observed comparing PPR values comparing all conditions: one-way ANOVA, F_(3,45)_ = 1.7, *p* = 0.2. NMDA current density values were not significantly different comparing all conditions: one-way ANOVA, F_(3,45)_ = 1.4, *p* = 0.2. NMDA/AMPA ratios were not significantly different comparing all conditions: one-way ANOVA, F_(3,31)_ = 0.8, *p* = 0.5.

**Table 3:** Quantitative PCR primers.

Gene	Gene Symbol	Primer Forward	Primer Reverse
*Beta Actin*	*Act B*	TGACGTTGACATCCGTAAAG	GAGGAGCAATGATCTTGATCT
*Neuronal PAS Domain Protein 4*	*Npas4*	CATCTGGGCCACTCTATGGT	GAGGGACTTGGAGGTGTTGA
*Early Growth Response 1*	*Egr1*	GATGGTGGAGACGAGTTAT	GATTGGTCATGCTCACG
*Fos Proto-Oncogene, AP-1 Transcription Factor Subunit*	*cFos*	TCCCCAAACTTCGACCATGA	AGTTGGCACTAGAGACGGAC
*Glutamate Ionotropic Receptor AMPA Type Subunit 1*	*GRIA1*	CTGTGAATCAGAACGCCTCA	TCACTTGTCCTCCACTGCTG
*Glutamate Ionotropic Receptor NMDA Type Subunit 2A*	*GRIN2A*	TTGTCTCTGCCATTGCTGTC	CAAAGAAGGCCCACACTGAT
*Serotonin Receptor 2A*	*Htr2a*	CGTGTCCATGTTAACCATCC	TCAGGAAGGCTTTGGTTCTG

## Data Availability

All data generated in the study are presented in the manuscript.

## Supplementary Materials

The following supporting information can be downloaded at: https://www.mdpi.com/article/10.3390/ijms25020687/s1.

## References

[1] Inserra A., De Gregorio D., Gobbi G. (2021). Psychedelics in Psychiatry: Neuroplastic, Immunomodulatory, and Neurotransmitter Mechanisms. Pharmacol. Rev..

[2] Raison C.L., Sanacora G., Woolley J., Heinzerling K., Dunlop B.W., Brown R.T., Kakar R., Hassman M., Trivedi R.P., Robison R. (2023). Single-Dose Psilocybin Treatment for Major Depressive Disorder: A Randomized Clinical Trial. JAMA.

[3] Mitchell J.M., Ot’alora G.M., van der Kolk B., Shannon S., Bogenschutz M., Gelfand Y., Paleos C., Nicholas C.R., Quevedo S., Balliett B. (2023). MAPP2 Study Collaborator Group. MDMA-assisted therapy for moderate to severe PTSD: A randomized, placebo-controlled phase 3 trial. Nat. Med..

[4] Bogenschutz M.P., Ross S., Bhatt S., Baron T., Forcehimes A.A., Laska E., Mennenga S.E., O’Donnell K., Owens L.T., Podrebarac S. (2022). Percentage of Heavy Drinking Days Following Psilocybin-Assisted Psychotherapy vs Placebo in the Treatment of Adult Patients With Alcohol Use Disorder: A Randomized Clinical Trial. JAMA Psychiatry.

[5] Shnayder S., Ameli R., Sinaii N., Berger A., Agrawal M. (2023). Psilocybin-assisted therapy improves psycho-social-spiritual well-being in cancer patients. J. Affect. Disord..

[6] Kwan A.C., Olson D.E., Preller K.H., Roth B.L. (2022). The neural basis of psychedelic action. Nat. Neurosci..

[7] Barre A., Berthoux C., De Bundel D., Valjent E., Bockaert J., Marin P., Bécamel C. (2016). Presynaptic serotonin 2A receptors modulate thalamocortical plasticity and associative learning. Proc. Natl. Acad. Sci. USA.

[8] Miner L.A.H., Backstrom J.R., Sanders-Bush E., Sesack S.R. (2003). Ultrastructural localization of serotonin 2A receptors in the middle layers of the rat prelimbic prefrontal cortex. Neuroscience.

[9] Weisstaub N.V., Zhou M., Lira A., Lambe E., González-Maeso J., Hornung J.P., Sibille E., Underwood M., Itohara S., Dauer W.T. (2006). Cortical 5-HT_2A_ receptor signaling modulates anxiety-like behaviors in mice. Science.

[10] Weber E.T., Andrade R. (2010). Htr2a Gene and 5-HT_2A_ Receptor Expression in the Cerebral Cortex Studied Using Genetically Modified Mice. Front. Neurosci..

[11] Lavaud C., Massiot G. (2017). The Iboga Alkaloids. Progress in the Chemistry of Organic Natural Products.

[12] González B., Fagundez C., Peixoto de Abreu Lima A., Suescun L., Sellanes D., Seoane G.A., Carrera I. (2021). Efficient Access to the Iboga Skeleton: Optimized Procedure to Obtain Voacangine from *Voacanga africana* Root Bark. ACS Omega.

[13] Iyer R.N., Favela D., Zhang G., Olson D.E. (2021). The iboga enigma: The chemistry and neuropharmacology of iboga alkaloids and related analogs. Nat. Prod. Rep..

[14] Obach R.S., Pablo J., Mash D.C. (1998). Cytochrome P4502D6 catalyzes the O-demethylation of the psychoactive alkaloid ibogaine to 12-hydroxyibogamine. Drug Metab. Dispos..

[15] Rodríguez P., Urbanavicius J., Prieto J.P., Fabius S., Reyes A.L., Havel V., Sames D., Scorza C., Carrera I. (2020). A Single Administration of the Atypical Psychedelic Ibogaine or Its Metabolite Noribogaine Induces an Antidepressant-Like Effect in Rats. ACS Chem. Neurosci..

[16] Alper K.R., Lotsof H.S., Frenken G.M., Luciano D.J., Bastiaans J. (1999). Treatment of acute opioid withdrawal with ibogaine. Am. J. Addict..

[17] Mash D.C., Kovera C.A., Pablo J., Tyndale R.F., Ervin F.D., Williams I.C., Singleton E.G., Mayor M. (2000). Ibogaine: Complex Pharmacokinetics, Concerns for Safety, and Preliminary Efficacy Measures. Ann. N. Y. Acad. Sci..

[18] Schenberg E.E., de Castro Comis M.A., Chaves B.R., da Silveira D.X. (2014). Treating drug dependence with the aid of ibogaine: A retrospective study. J. Psychopharmacol..

[19] Mash D.C., Duque L., Page B., Allen-Ferdinand K. (2018). Ibogaine Detoxification Transitions Opioid and Cocaine Abusers Between Dependence and Abstinence: Clinical Observations and Treatment Outcomes. Front. Pharmacol..

[20] Köck P., Frölich K., Walter M., Lang U., Dürsteler K.M. (2021). A systematic literature review of clinical trials and therapeutic applications of ibogaine. J. Subst. Abuse Treat..

[21] Wasko M.J., Witt-Enderby P.A., Surratt C.K. (2018). DARK Classics in Chemical Neuroscience: Ibogaine. ACS Chem. Neurosci..

[22] Mash D.C., Staley J.K., Pablo J.P., Holohean A.M., Hackman J.C., Davidoff R.A. (1995). Properties of ibogaine and its principal metabolite (12-hydroxyibogamine) at the MK-801 binding site of the NMDA receptor complex. Neurosci. Lett..

[23] Layer R.T., Skolnick P., Bertha C.M., Bandarage U.K., Kuehne M.E., Popik P. (1996). Structurally modified ibogaine analogs exhibit differing affinities for NMDA receptors. Eur. J. Pharmacol..

[24] Staley J.K., Ouyang Q., Pablo J., Hearn W.L., Flynn D.D., Rothman R.B., Rice K.C., Mash D.C. (1996). Pharmacological screen for activities of 12-hydroxyibogamine: A primary metabolite of the indole alkaloid ibogaine. Psychopharmacology.

[25] Glue P., Cape G., Tunnicliff D., Lockhart M., Lam F., Hung N., Hung C.T., Harland S., Devane J., Crockett R.S. (2016). Ascending Single-Dose, Double-Blind, Placebo-Controlled Safety Study of Noribogaine in Opioid-Dependent Patients. Clin. Pharmacol. Drug Dev..

[26] Tyler M.W., Yourish H.B., Ionescu D.F., Haggarty S.J. (2017). Classics in Chemical Neuroscience: Ketamine. ACS Chem. Neurosci..

[27] Hernández-Alvarado R.B., Madariaga-Mazón A., Ortega A., Martinez-Mayorga K. (2020). DARK Classics in Chemical Neuroscience: Salvinorin A. ACS Chem. Neurosci..

[28] Helsley S., Fiorella D., Rabin R.A., Winter J.C. (1998). Behavioral and biochemical evidence for a nonessential 5-HT_2A_ component of the ibogaine-induced discriminative stimulus. Pharmacol. Biochem. Behav..

[29] González J., Prieto J.P., Rodríguez P., Cavelli M., Benedetto L., Mondino A., Pazos M., Seoane G., Carrera I., Scorza C. (2018). Ibogaine Acute Administration in Rats Promotes Wakefulness, Long-Lasting REM Sleep Suppression, and a Distinctive. Motor Profile. Front. Pharmacol..

[30] González J., Cavelli M., Castro-Zaballa S., Mondino A., Tort A.B., Rubido N., Carrera I., Torterolo P. (2021). EEG Gamma Band Alterations and REM-like Traits Underpin the Acute Effect of the Atypical Psychedelic Ibogaine in the Rat. ACS Pharmacol. Transl. Sci..

[31] Ly C., Greb A.C., Cameron L.P., Wong J.M., Barragan E.V., Wilson P.C., Burbach K.F., Zarandi S.S., Sood A., Paddy M.R. (2018). Psychedelics Promote Structural and Functional Neural Plasticity. Cell Rep..

[32] Mauvais-Jarvis F., Merz N.B., Barnes P.J., Brinton R.D., Carrero J.J., DeMeo D.L., De Vries G.J., Epperson C.N., Govindan R., Klein S.L. (2020). Sex and gender: Modifiers of health, disease, and medicine. Lancet.

[33] Pearl S.M., Hough L.B., Boyd D.L., Glick S.D. (1997). Sex differences in ibogaine antagonism of morphine-induced locomotor activity and in ibogaine brain levels and metabolism. Pharmacol. Biochem. Behav..

[34] Tatalović N., Vidonja Uzelac T., Mijović M., Koželj G., Nikolić-Kokić A., Oreščanin Dušić Z., Bresjanac M., Blagojević D. (2021). Ibogaine Has Sex-Specific Plasma Bioavailability, Histopathological and Redox/Antioxidant Effects in Rat Liver and Kidneys: A Study on Females. Life.

[35] Glick S.D., Pearl S.M., Cai J., Maisonneuve I.M. (1996). Ibogaine-like effects of noribogaine in rats. Brain Res..

[36] Maillet E.L., Milon N., Heghinian M.D., Fishback J., Schürer S.C., Garamszegi N., Mash D.C. (2015). Noribogaine is a G-protein biased κ-opioid receptor agonist. Neuropharmacology.

[37] Kuzmin A., Sandin J., Terenius L., Ogren S.O. (2000). Dose- and time-dependent bimodal effects of kappa-opioid agonists on locomotor activity in mice. J. Pharmacol. Exp. Ther..

[38] Yuen E.Y., Jiang Q., Chen P., Feng J., Yan Z. (2008). Activation of 5-HT_2A_/C receptors counteracts 5-HT1A regulation of n-methyl-D-aspartate receptor channels in pyramidal neurons of prefrontal cortex. J. Biol. Chem..

[39] Jaggar M., Banerjee T., Weisstaub N., Gingrich J.A., Vaidya V.A. (2019). 5-HT_2A_ receptor loss does not alter acute fluoxetine-induced anxiety and exhibit sex-dependent regulation of cortical immediate early gene expression. Neuronal Signal..

[40] González B., Rivero-Echeto C., Muñiz J.A., Cadet J.L., García-Rill E., Urbano F.J., Bisagno V. (2016). Methamphetamine blunts Ca^2+^ currents and excitatory synaptic transmission through D1/5 receptor-mediated mechanisms in the mouse medial prefrontal cortex. Addict. Biol..

[41] Myme C.I.O., Sugino K., Turrigiano G.G., Nelson S.B. (2003). The NMDA-to-AMPA ratio at synapses onto layer 2/3 pyramidal neurons is conserved across prefrontal and visual cortices. J. Neurophysiol..

[42] Carhart-Harris R.L., Erritzoe D., Williams T., Stone J.M., Reed L.J., Colasanti A., Tyacke R.J., Leech R., Malizia A.L., Murphy K. (2012). Neural correlates of the psychedelic state as determined by fMRI studies with psilocybin. Proc. Natl. Acad. Sci. USA.

[43] Higgins G.A., Carroll N.K., Brown M., MacMillan C., Silenieks L.B. (2021). Low Doses of Psilocybin and Ketamine Enhance Motivation and Attention in Poor Performing Rats: Evidence for an Antidepressant Property. Front. Pharmacol..

[44] Leslie J.H., Nedivi E. (2011). Activity-regulated genes as mediators of neural circuit plasticity. Prog. Neurobiol..

[45] Veyrac A., Besnard A., Caboche J., Davis S., Laroche S. (2014). The transcription factor Zif268/Egr1, brain plasticity, and memory. Prog. Mol. Biol. Transl. Sci..

[46] Spiegel I., Mardinly A.R., Gabel H.W., Bazinet J.E., Couch C.H., Tzeng C.P., Harmin D.A., Greenberg M.E. (2014). Npas4 regulates excitatory-inhibitory balance within neural circuits through cell-type-specific gene programs. Cell.

[47] Maya-Vetencourt J.F., Tiraboschi E., Greco D., Restani L., Cerri C., Auvinen P., Maffei L., Castrén E. (2012). Experience-dependent expression of NPAS4 regulates plasticity in adult visual cortex. J. Physiol..

[48] Zhang G., Stackman R.W. (2015). The role of serotonin 5-HT_2A_ receptors in memory and cognition. Front. Pharmacol..

[49] Aznar S., Hervig M.E.S. (2016). The 5-HT_2A_ serotonin receptor in executive function: Implications for neuropsychiatric and neurodegenerative diseases. Neurosci. Biobehav. Rev..

[50] DeBattista C. (2005). Executive dysfunction in major depressive disorder. Expert Rev. Neurother..

[51] Qesseveur G., Petit A.C., Nguyen H.T., Dahan L., Colle R., Rotenberg S., Seif I., Robert P., David D., Guilloux J.P. (2015). Genetic dysfunction of serotonin 2A receptor hampers response to antidepressant drugs: A translational approach. Neuropharmacology.

[52] Popik P., Layer R.T., Fossom L.H., Benveniste M., Geter-Douglass B., Witkin J.M., Skolnick P. (1995). NMDA antagonist properties of the putative antiaddictive drug, ibogaine. J. Pharmacol. Exp. Ther..

[53] Chen K., Kokate T.G., Donevan S.D., Carroll F.I., Rogawski M.A. (1996). Ibogaine block of the NMDA receptor: In vitro and in vivo studies. Neuropharmacology.

[54] Castren E., Antila H. (2017). Neuronal plasticity and neurotrophic factors in drug responses. Mol. Psychiatry.

[55] Rantamäki T., Hendolin P., Kankaanpää A., Mijatovic J., Piepponen P., Domenici E., Chao M.V., Männistö P.T., Castrén E. (2007). Pharmacologically diverse antidepressants rapidly activate brain-derived neurotrophic factor receptor TrkB and induce phospholipase-C gamma signaling pathways in mouse brain. Neuropsychopharmacology.

[56] Popova N.K., Ilchibaeva T.V., Naumenko V.S. (2017). Neurotrophic Factors (BDNF and GDNF) and the Serotonergic System of the Brain. Biochemistry.

[57] Marton S., González B., Rodríguez-Bottero S., Miquel E., Martínez-Palma L., Pazos M., Prieto J.P., Rodríguez P., Sames D., Seoane G. (2019). Ibogaine Administration Modifies GDNF and BDNF Expression in Brain Regions Involved in Mesocorticolimbic and Nigral Dopaminergic Circuits. Front. Pharmacol..

[58] Pollina E.A., Gilliam D.T., Landau A.T., Lin C., Pajarillo N., Davis C.P., Harmin D.A., Yap E.L., Vogel I.R., Griffith E.C. (2023). A NPAS4-NuA4 complex couples synaptic activity to DNA repair. Nature.

[59] Petit A.C., Quesseveur G., Gressier F., Colle R., David D.J., Gardier A.M., Ferreri F., Lépine J.P., Falissard B., Verstuyft C. (2014). Converging translational evidence for the involvement of the serotonin 2A receptor gene in major depressive disorder. Prog. Neuropsychopharmacol. Biol. Psychiatry.

[60] Covington HE 3rd Vialou V., Nestler E.J. (2010). From synapse to nucleus: Novel targets for treating depression. Neuropharmacology.

[61] Koenigs M., Grafman J. (2009). The functional neuroanatomy of depression: Distinct roles for ventromedial and dorsolateral prefrontal cortex. Behav. Brain Res..

[62] Lefaucheur J.P., Antal A., Ayache S.S., Benninger D.H., Brunelin J., Cogiamanian F., Cotelli M., De Ridder D., Ferrucci R., Langguth B. (2017). Evidence-based guidelines on the therapeutic use of transcranial direct current stimulation (tDCS). Clin. Neurophysiol..

[63] Diazgranados N., Ibrahim L., Brutsche N.E., Newberg A., Kronstein P., Khalife S., Kammerer W.A., Quezado Z., Luckenbaugh D.A., Salvadore G. (2010). A randomized add-on trial of an N-methyl-D-aspartate antagonist in treatment-resistant bipolar depression. Arch. Gen. Psychiatry.

[64] Stasiuk W., Szopa A., Serefko A., Wyska E., Świąder K., Dudka J., Wlaź P., Poleszak E. (2017). Influence of the selective antagonist of the NR2B subunit of the NMDA receptor, traxoprodil, on the antidepressant-like activity of desipramine, paroxetine, milnacipran, and bupropion in mice. J. Neural Transm..

[65] Bisagno V., Raineri M., Peskin V., Wikinski S.I., Uchitel O.D., Llinás R.R., Urbano F.J. (2010). Effects of T-type calcium channel blockers on cocaine-induced hyperlocomotion and thalamocortical GABAergic abnormalities in mice. Psychopharmacology.

