# Supplementary material for: 5-HT2A Receptor Knockout Mice Show Sex-Dependent Differences following Acute Noribogaine Administration
Source: Int J Mol Sci. 2024 Jan 5;25(2):687. doi: 10.3390/ijms25020687 (PMC10815577; doi:10.3390/ijms25020687)
Supplement: Supplementary file 1 [file ijms-25-00687-s001.zip › ijms-2785934-supplementary.pdf]

Supplementary Information

Supplementary Figure S1: Patch clamp recordings during paired-pulse stimulation. AMPA and NMDA-mediated components of evoked postsynaptic currents (EPSCs)

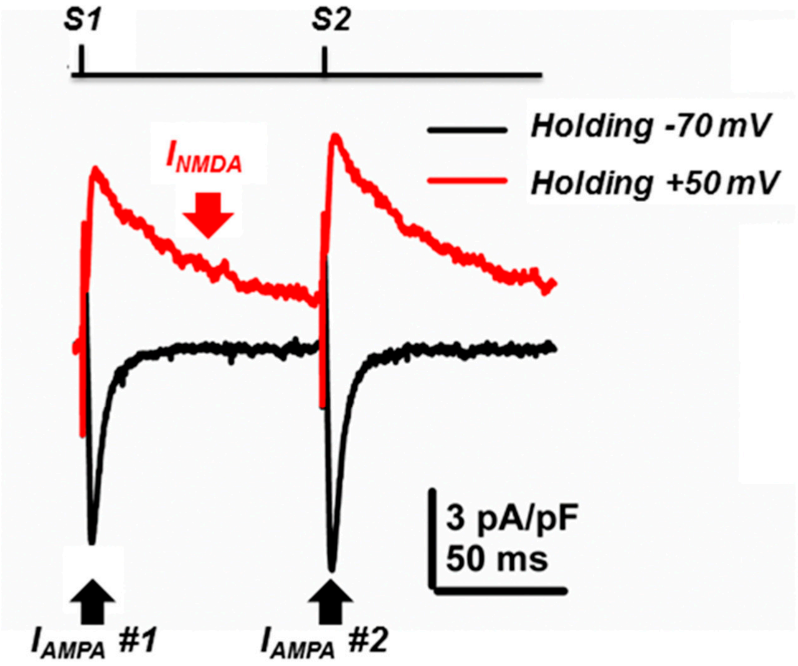

$$\text{Paired-Pulse Ratio (PPR)} = \frac{I_{AMPA}\ \#2}{I_{AMPA}\ \#1}$$

Figure S1: Representative paired-pulse (See above, S1 & S2 stimuli, separated 100 ms) evoked excitatory postsynaptic currents (EPSC) obtained using whole-cell patch-clamp recordings were performed in medial prefrontal coronal slices in an extracellular ACSF solution containing $MgCl_2$ (1 mM), $CaCl_2$ (1 mM) and bicuculline (5- 10 μM), as previously described (ref, #40). Paired pulse ratio (PPR) shown in figures 4 and 5 were calculated as the fraction of 2nd AMPA-mediated EPSC (I AMPA #2)/1st (I AMPA #1) peak amplitudes (black arrowheads) while pyramidal cells were held at -70 mV holding (black trace). NMDA-mediated current density was measured 50 ms after first stimulus artifact at +50 mV holding, a fraction of currents that was blocked in the presence of AP5 (50 μM), as previously described (ref. #41).

Supplementary Figures S2 & S3: Noribogaine characterization

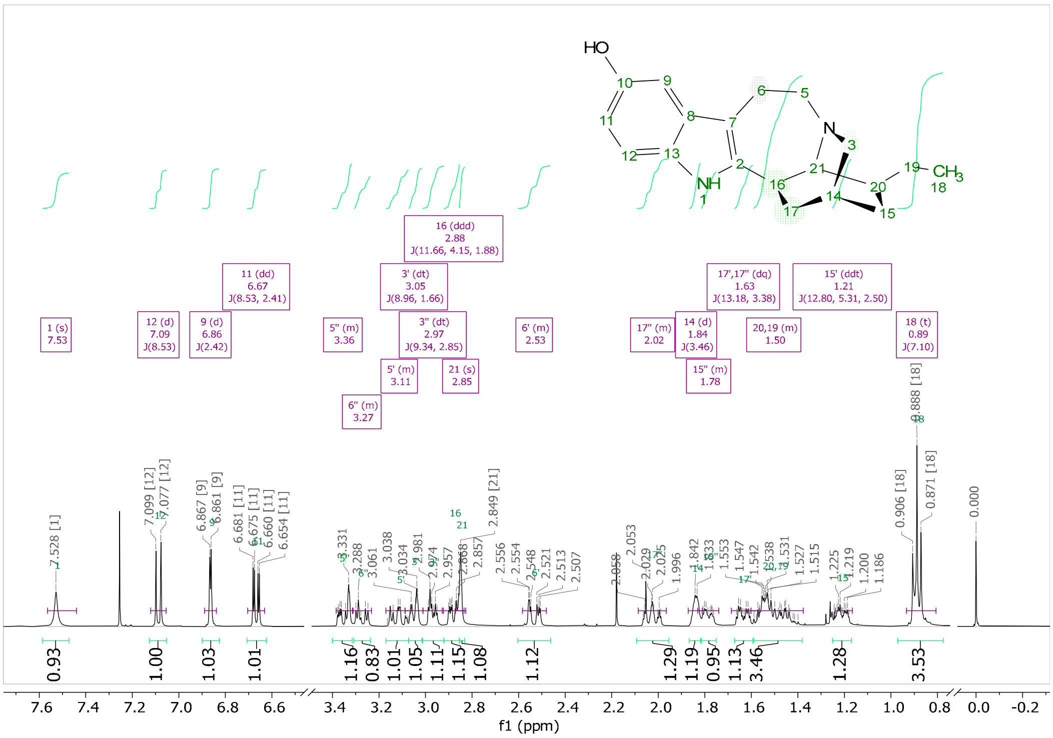

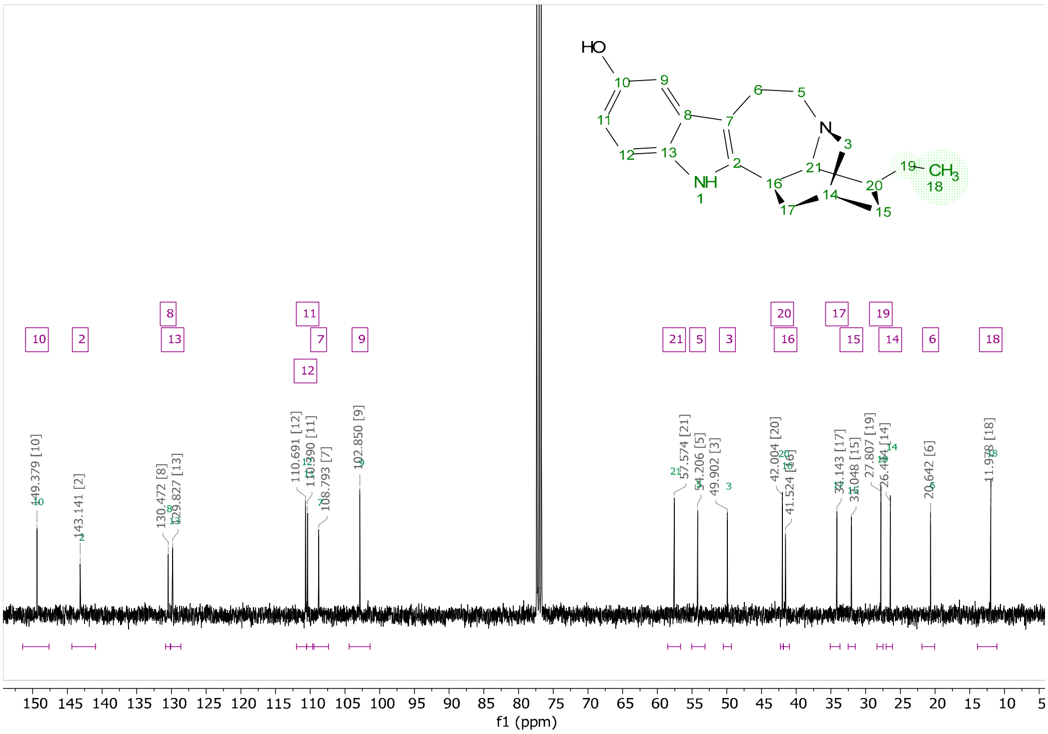

Figure S2: **^{1}H-NMR and ^{13}C-NMR spectra for noribogaine.** The material obtained was analyzed using Nuclear Magnetic Resonance. Full characterization and assignments are shown in the corresponding Figures. NMR spectra were obtained in $CDCl_3$ on a Bruker Avance Neo 400 instrument operating at a ^{1}H frequency of 400 MHz. Proton chemical shifts (δ) are reported in

ppm downfield from TMS (0.000 ppm) as an internal reference, and carbon chemical shifts are reported in ppm relative to the center line of the $CDCl_3$ triplet (77.0 ppm).

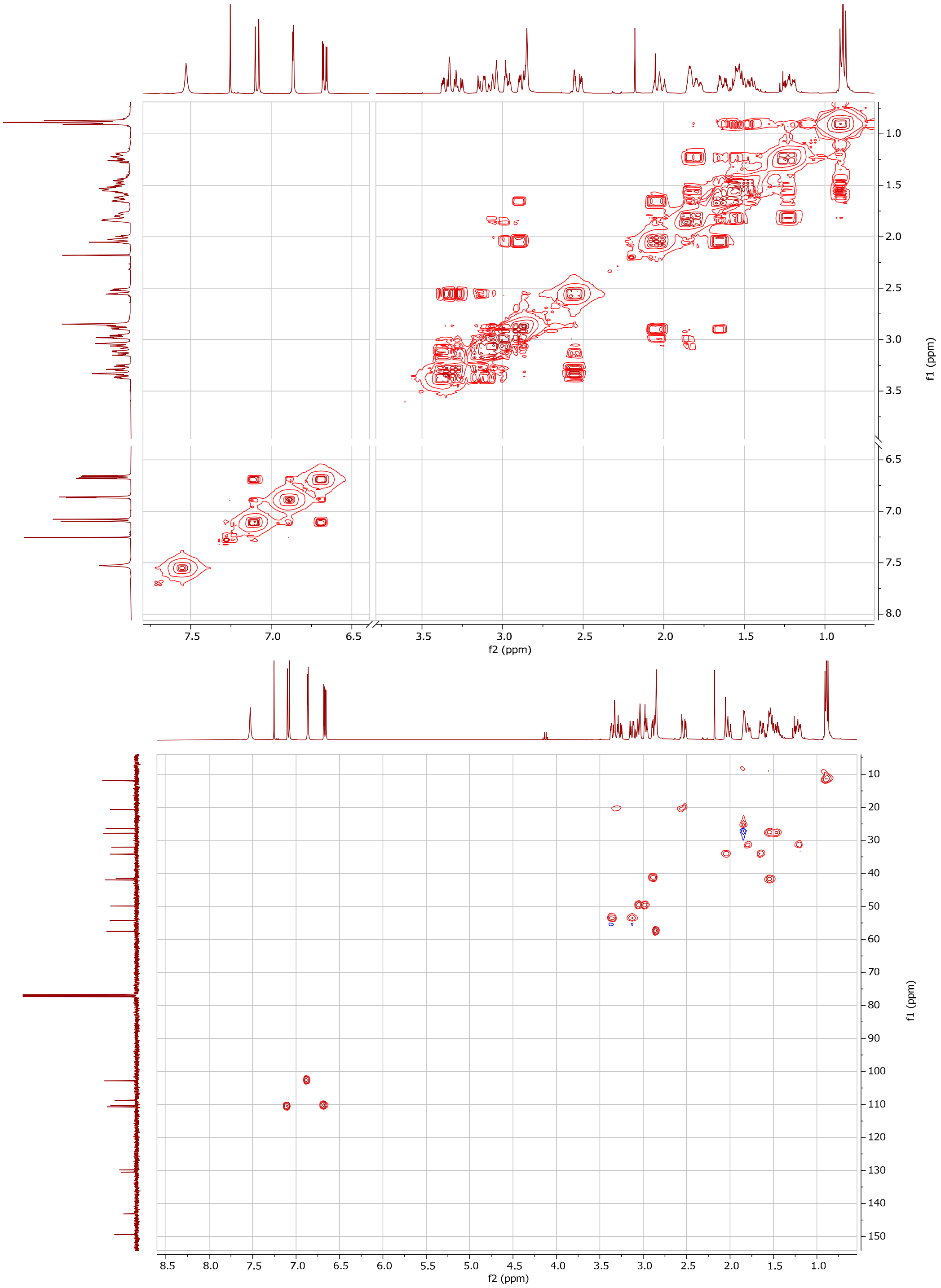

Figure S3: **Bidimensional spectra used for characterization and H and C assignment of noribogaine.** COSY and HSQC used for the elucidation of the chemical structure of noribogaine are provided.

Supplementary Information of primers' sequences used for genotyping

2A PCR Geno upper CTG TGG GAT TTT CTT TCT GCT

2A PCR Geno lower GTG TGA TGG CTC TTG ATT ATG C

2A PCR Geno tta lower TGG GCG AGT TTA CGG GTT GTT A
